# Initial treatment with a single capsule containing half-dose quadruple therapy versus standard-dose dual therapy in hypertensive patients (QUADUAL): statistical analysis plan for a randomized, blinded, crossover trial

**DOI:** 10.1186/s13063-023-07803-1

**Published:** 2024-01-13

**Authors:** Xiexiong Zhao, Xingli Li, Tao Liu, Guoping Yang, Ye Chen, Miao Huang, Lin Zhao, Xiaogang Li, Weihong Jiang

**Affiliations:** 1 Department of Cardiology, The Third Xiangya Hospital of Central South University, 138 Tongzipo Road, Yuelu District, Changsha, 410013 Hunan China; 2 Department of Epidemiology, XiangYa School of Public Health, Changsha, China; 3 Center of Clinical Pharmacology, The Third Xiangya Hospital of Central South University, 138 Tongzipo Road, Yuelu District, Changsha, 410013 Hunan China; 4 Department of Clinical Pharmacology, XiangYa School of Pharmaceutical Sciences, Changsha, China; 5 Hypertension Research Center of Hunan Province, Changsha, Hunan China

**Keywords:** Hypertension, Statistical analysis plan, Antihypertensive, Low-dose combination, Randomized controlled trial

## Abstract

**Background:**

Combined antihypertensive therapy has obvious advantages over single drug therapy. Hypertension guidelines fully affirm the efficacy of dual combination in initial antihypertensive therapy. Recent studies have also pointed out that the quadruple combination of very low-dose antihypertensive drugs is superior to single drugs. However, whether low-dose quadruple therapy is better than dual combination is unknown.

**Methods/design:**

A randomized double-blind crossover clinical trial will be conducted to compare the efficacy and safety of low-dose quadruple antihypertensives (irbesartan 75 mg + metoprolol 23.75 mg + amlodipine 2.5 mg + indapamide 1.25 mg) with standard-dose dual antihypertensives (irbesartan 150 mg + amlodipine 5 mg) in the initial treatment of patients with mild to moderate hypertension (140–179/90–109 mmHg). Ninety patients are required and will be recruited and randomly assigned in a 1:1 ratio to two crossover groups. Two groups will receive a different combination therapy for 4 weeks, then switch to the other combination therapy for 4 weeks, with a 2-week wash-out. Antihypertensive effects and related adverse effects of the two antihypertensive combination treatments will be compared. The primary outcome, i.e., mean 24-h systolic blood pressure in ambulatory blood pressure monitoring, will be assessed via linear mixed-effects model.

**Discussion:**

This statistical analysis plan will be confirmed prior to blind review and data lock before un-blinding and is sought to increase the validity of the QUADUAL trial.

**Trial registration:**

ClinicalTrials.gov, NCT05377203. Registered May 11, 2022, https://clinicaltrials.gov/study/NCT05377203.

## Introduction

### Background and rationale

Hypertension is the most common cardio-cerebrovascular disease worldwide, with a significant population affected, substantial health risks, and a heavy economic burden [1–4]. However, the awareness, treatment, and control rates of hypertension remain suboptimal, with data from China indicating rates of only 50.0%, 38.1%, and 11.1%, respectively [5].

Current hypertension guidelines have recognized the efficacy of dual combination therapy as an initial antihypertensive treatment [1, 6–11]. However, hypertension involves multiple mechanisms [12, 13], and the goal of blood pressure control has become more stringent. As a result, dual combination therapy may not be sufficient to meet the needs of patients. Consequently, some researchers have explored the use of low-dose three-drug or four-drug combinations [14–18]. However, these studies employed monotherapy or placebo as controls, which are not consistent with current guidelines for initial hypertension treatment. Furthermore, these studies did not demonstrate whether low-dose multidrug (≥ 3) combinations were more effective than the current recommended dual combinations, and none of these studies included Chinese population. Therefore, this trial will be the first to investigate the effectiveness and safety of low-dose quadruple combination therapy compared to dual combination therapy in the Chinese population.

### Objective

The objective is as follows: to evaluate and compare the efficacy and safety of half-dose quadruple therapy versus standard-dose dual therapy in the initial treatment of hypertensive patients with mild to moderate blood pressure (140–179/90–109 mmHg).

## Study methods

### Trial design

This is a randomized, double-blind, two-agent, two-cycle, two-sequence crossover clinical trial, comparing the effectiveness and safety of low-dose quadruple antihypertensives (irbesartan 75 mg + metoprolol 23.75 mg + amlodipine 2.5 mg + indapamide 1.25 mg) with standard-dose dual drugs (irbesartan 150 mg + amlodipine 5 mg) in initial antihypertensive treatment in patients with mild to moderate hypertension (140–179/90–109 mmHg). We will enroll 90 patients in the Third Xiangya Hospital of Central South University. The design of this trial has been described in detail in our protocol for this trial [19]. This statistical analysis plan (SAP) was written following the guidelines for the content of statistical analysis plans in clinical trials [20].

### Randomization and blinding

In this trial, stratified blocked randomization and individual random crossover will be adopted to minimize the influence of seasonal and temperature changes on the results, dividing participants into 2 crossover groups in a 1:1 ratio. Randomization and blinding will be established by an independent statistician.

Except for randomizing, blinding, and drug coding investigators, all others (including participants, clinical investigators, coordinators, clinical research associates, all members of the Clinical Endpoint Committee (CEC), data managers, statistical analysts, drug manufacturers, and administration) are blinded to patient grouping and drug assignment.

This trial will use two-time unblinding method. When the data file is confirmed and locked, the first unblinding will be performed, which only lists the group to which each case belongs for analysis (such as group A or group B). After the statistical analysis is complete, the second unblinding will be performed to determine which treatment option is used in the two groups.

### Sample size

In the 2021 QUARTET study [18], the 1/4 dose quadruple combination (irbesartan 37.5 mg, amlodipine 1.25 mg, indapamide 0.625 mg, and bisoprolol 2.5 mg) further reduced systolic blood pressure (SBP) by 6.9 mmHg (95% CI 4.9–8.9) compared to single drug (irbesartan 150 mg), with an estimated standard deviation (SD) of 15 mmHg.

At the same time, based on the previous clinical observation results of the research group on low-dose quadruple combination and standard-dose dual combination, it is estimated that the difference in 24-h mean SBP reduction between the two groups is 6 mmHg, with an SD 15 mmHg. Power is set at 90% (beta = 0.1) and an acceptable risk of type I error is 5% (two-sided alpha level).

We use the following formula, which is specially for sample size calculation of cross-over design, [21] to calculate the total number:

$${n = [\frac{({t}_{\alpha }+{t}_{2\beta })S}{\delta }]^{2}}$$

The result is *n* = 66. And we also calculate the sample size via PASS 11.0 (Power Analysis & Sample Size, NCSS, LLC.) (for 2 × 2 cross-over design) with the result *n* = 68. Taking the larger one and considering 20% loss to follow-up, 85 participants are calculated, and considering the random factors of the block group, a final sample size of 90 participants with 45 in each crossover group is needed.

### Data monitoring

This trial will establish an independent data monitoring committee (IDMC) to report to the clinical trial research center and ethics committee. The purpose of the IDMC is to protect the safety of the participants, ensure the validity of the data, and decide the timely termination of the trial when a significant benefit or risk is demonstrated or a successful conclusion is impossible. The IDMC will be responsible for assessing the safety of therapeutic interventions during the study period, thereby protecting the interests of patients, and for reviewing the overall conduct of the clinical trial.

### Timing of final analysis

All outcomes will be analyzed collectively after data entry and data monitoring have been completed and the database has been cleaned and closed.

### Statistical principles

#### Confidence intervals and *P* values

In this study, *P* < 0.05 will be considered statistically significant and 95% confidence interval will be reported if applicable.

#### Adherence and protocol deviations

Medication compliance = (total number of pills issued—number of pills recovered)/days of medication × 100%. Medication compliance will be demonstrated. Medication compliance of 80–120% will be considered as condition of per-protocol set (PPS).

#### Analysis populations

According to the principle of intention to treat (ITT), there are three analysis populations involved in this study: the full analysis set (FAS), PPS, and the safety set (SS). The definitions of each analysis set are given below:

##### FAS

All cases that do not violate the main inclusion/exclusion criteria, use the drug at least once after randomization, and have at least 1 post-dose efficacy evaluation data will be considered as the FAS for the analysis of efficacy. For those who do not complete treatment as planned, the last observation will be used as the final outcome (last observation carried forward, LOCF).

##### PPS

It is the subset of the FAS that is more compliant with the protocol. These participants are more adherent to the protocol. Individuals in the PPS are required to meet the following characteristics:

- Medication compliance is 80–120%;
- Treatment meets efficacy endpoints as protocol required, and the primary outcomes are measurable;
- No major violations of the protocol (including inclusion and exclusion criteria).

##### SS

All participants who use the drug at least once after randomization are part of this subset.

#### Trial population

**Fig. 1:**
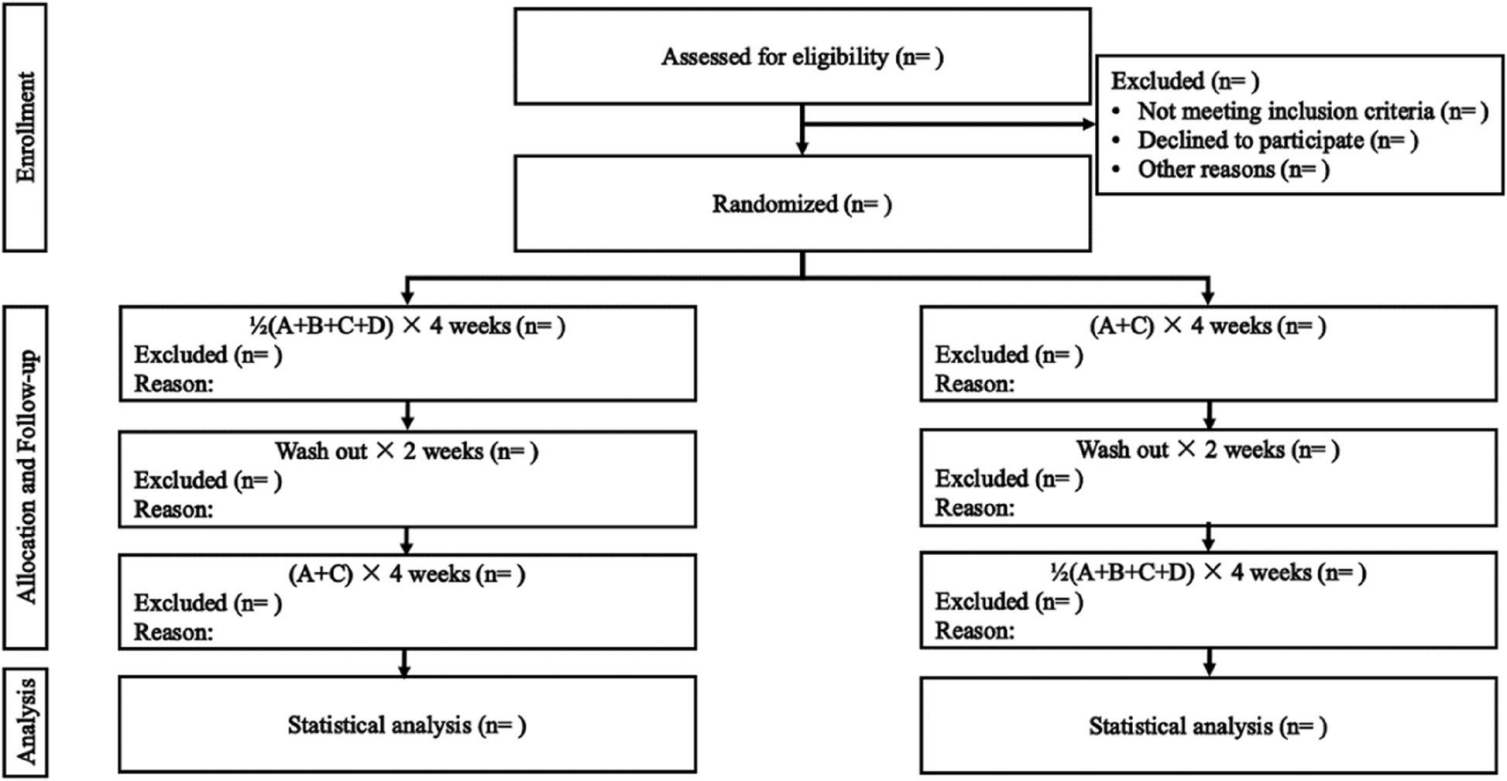
Flow diagram of the QUADUAL trial. **A** Angiotensin receptor blocker (irbesartan 150 mg). **B** Beta-blocker (metoprolol 47.5 mg). **C** Calcium channel blocker (amlodipine 5 mg). **D** Diuretic (indapamide 2.5 mg)

All hypertensive patients who have never taken antihypertensive medications or have not taken antihypertensive medications in the past 1 month will be eligible and screened consecutively with inclusion and exclusion criteria in the department of cardiology at the Third Xiangya Hospital, Central South University. A Consolidated Standards of Reporting Trials (CONSORT) flow diagram (Fig. 1) will be produced according to CONSORT 2010 Statement [22].

#### Demographic and baseline characteristics

Demographic and baseline characteristics will be descriptively tabulated and summarized for all subjects in FAS. For continuous variables, the mean and SD (normal distribution) or median and 25th/75th percentile (non-normal distribution) will be given. For categorical variables, the number and percentage of subjects will be given.

### Analysis

#### Outcome definitions

##### Primary outcome

The primary outcome is established as the reduction in mean 24-h SBP by ambulatory blood pressure monitoring (ABPM) after 4 weeks of drug administration.

##### Secondary outcomes

- Mean daytime and nighttime SBP in ABPM, change from baseline
- 24-h, daytime, and nighttime mean diastolic blood pressure (DBP) in ABPM, change from baseline
- Morning BP surge in ABPM, change from baseline
- Office blood pressure measurement (OBPM), change from baseline
- Home blood pressure measurement (HBPM), change from baseline
- Heart rate, change from baseline
- Blood pressure control rate

##### Safety outcomes

The safety outcomes are as follows: adverse event (AE), serious adverse event (SAE), adverse drug reaction (ADR), and changes in biochemistry results and QT interval of the electrocardiogram from baseline.

Timings of outcome assessments are listed in Table 1. Blood pressure measurement methods (including ABPM, OBPM, and HBPM) were detailed in the previously published protocol [19]. For HBPM, 4–6 BP data will be recorded in “Patient Manual” by participants according to the agreement. The average of all the BPs for 1 day will be used as the BP value for that day. HBPM on the day before the follow-up visit will be used as the HBPM at the end of this period.

**Table 1:** Timing of outcome assessments

**Timepoint**	**Enrolment and allocation**	**Treatment phase 1**		**Washout phase**		**Treatment phase 2**	
	**0 day**	**1–4 weeks**	**4th week**	**5–6 weeks**	**6th week**	**7–10 weeks**	**10th week**
HBPM	X	X	X	X	X	X	X
OBPM	X		X		X		X
Biochemistry results							
Electrolyte	X		X				X
FBG	X		X				X
Renal function	X		X				X
Liver function	X		X				X
Urine routine	X		X				X
Tests							
ECG	X		X				X
ABPM	X		X		X		X

*ABMP* ambulatory blood pressure monitoring, *ECG* electrocardiogram, *FBG* fasting blood-glucose, *HBPM* home blood pressure measurement, *OBPM* office blood pressure measurement

#### Criteria for blood pressure control

ABPM: 24-h average blood pressure < 130/80 mmHg; daytime average blood pressure < 135/85 mmHg; nighttime average blood pressure < 120/70 mmHg.

OBPM: SBP/DBP < 140/90 mmHg.

HBPM: SBP/DBP < 135/85 mmHg; time in target range (TTR) of HBPM = days meet target/days of medication × 100%.

#### Definition of baseline

This crossover trial includes three phases: treatment phase 1 (weeks 1–4), washout phase (weeks 5–6), and treatment phase 2 (weeks 6–10). The baselines of treatment phase 1 are defined as the results obtained from enrolment period, including all primary, secondary, and safety indicators. The baselines of treatment phase 2 are defined as the results obtained from the end of the washout phase (for ABMP, OBPM, and HBPM) and enrolment period (for the rest of the indicators).

#### Statistical hypothesis

For this exploratory study, the following hypotheses will be used for the primary outcome:

$$\mathrm{Original\ hypotheses\ }{H}_{0}\!\! :\ {\mu }_{T}={\mu }_{C};\ \mathrm{alternative\ hypotheses\ }{H}_{1}\!\! :\ {\mu }_{T}\ne {\mu }_{C}$$

where ${\mu }_{T}$ is for the mean effect of half-dose quadruple therapy, and ${\mu }_{C}$ is for the mean effect of standard-dose dual therapy.

#### Analysis of primary outcome

PPS will be mainly used for analysis of primary outcome. Linear mixed-effects model will be used to analyze treatment effects, stage effects, and order effects (residual carryover effect) [23, 24]. In this model, treatment, group, and stage will be the fixed effects, baseline blood pressure will be the covariates, and subjects will be the random effects. The model is as follows:

$${Y}_{\text{ijtk}}=\mu +{\gamma }_{i}+{\pi }_{j}+{\sigma }_{t}+{S}_{k(t)}+{\varepsilon }_{\text{ijtk}}$$

where *i* is the group (2 crossover groups, 0 or 1), *j* is the number of stages (2 stages, 1 or 2), *t* is the drug (2 drugs, 0 and 1), and *k* represents the individual (90 subjects). *Y*_ijtk_ is the observed trial effect (mean SBP reduction in ABPM after 4 weeks of drug administration) for the *k* th subject in group *i*, at phase *j*, and with drug *t*. $\mu$ is the overall mean effect, ${\gamma }_{i}$ is the fixed effect for group *i*, ${\pi }_{j}$ is the fixed effect for the *j* th stage, ${\sigma }_{t}$ is the fixed effect for the *t* th drug, ${S}_{k(t)}$ is the random effect for the *k* th subject with the *t* th drug, ${\varepsilon }_{\text{ijtk}}$ is the residual of *Y*_ijtk_, or random error.

On the basis of the above model, baseline characteristics such as age, gender, nationality, time of hypertension, smoking, alcohol, body mass index, waistline, diabetes, and estimated glomerular filtration rate will be corrected to construct an adjusted model.

#### Analysis of secondary outcomes

PPS will be used for analysis of secondary outcomes. Measurement data (changes of blood pressure and pulse rate, TTR, etc.) will be analyzed using the linear mixed-effects model described above, and counting data (blood pressure control rate) will be analyzed using the paired chi-square test or Fisher’s exact probability methods.

#### Analysis of safety outcomes

SS will be used for analysis of safety outcomes.

The incidence of AEs, SAEs, and ADRs will be summarized by system and organ, counted in terms of number, severity, and relationship to each therapeutic drug, which will be compared between the two medications using chi-square tests or Fisher’s exact probability method.

Changes in biochemistry results and QT interval of the electrocardiogram will be analyzed using linear mixed-effects model. The incidence of concerned abnormal values (including hypokalemia; hyponatremia; serum creatinine, uric acid, urea, ALT, AST, TBL, DBL, blood glucose, QT and QTc elevated above the upper limit of normal (ULN), etc.) will be summarized and analyzed using chi-square test or Fisher’s exact probability method. Analysis methods for different outcomes are list in Table 2.

**Table 2:** Analysis methods for different outcomes

Outcomes	Analysis methods
**Primary outcome**	
Changes in 24-h SBP	Linear mixed-effects model
**Secondary outcomes**	
Changes in 24-h DBP	Linear mixed-effects model
Changes in daytime BP	Linear mixed-effects model
Changes in nighttime BP	Linear mixed-effects model
Changes in morning BP surge	Linear mixed-effects model
Changes in office BP	Linear mixed-effects model
Changes in home BP	Linear mixed-effects model
Changes in heart rate	Linear mixed-effects model
BP control rate	Paired chi-square test
TTR of home BP	Linear mixed-effects model
**Safety outcomes**	
Adverse event	Chi-square tests or Fisher’s exact probability method
Changes in biochemistry results	Linear mixed-effects model
Changes in QT interval	Linear mixed-effects model

*BP* blood pressure, *DBP* diastolic blood pressure, *SBP* systolic blood pressure, *TTR* time in target range

#### Sensitivity analysis

Sensitivity analysis will be conducted in the following situations:

- FAS for analysis of primary and secondary outcomes;
- Different ways of managing missing data for analysis of HBPM;
- Retention or exclusion of outliers if applicable.

#### Subgroup analysis

Subgroup analysis will be performed based on the following situation:

- Sex (male or female)
- Age (< 45 years or ≥ 45years, which is used to classify youth and middle age)
- Diabetes mellitus (with or without)

#### Handling of missing data

We will impute missing data of HBPM using LOCF method. Sensitivity analysis will use multiple imputation. For analyses of primary and remaining secondary outcomes, imputation will not be used.

#### Handling of outliers

Outliers, if applicable, will not be excluded while a sensitivity analysis will be conducted with or without outliers.

#### Statistical software

All statistical analyses will be performed by statistician using IBM SPSS Statistics Version 23 and RStudio 2023.06.0 + 421.

## Trial status

The trial was initiated on July 4, 2022, in the Third Xiangya Hospital of Central South University. The trial began enrolling on July 13, 2022, finished enrolling on April 20, 2023, and finished last participant’s last visit on July 4, 2023. Data entry is currently in progress. We anticipate blind review and database lock to be conducted by the end of August, 2023.

## SAP version

Version 1.0 (dated July 25, 2023) based on QUADUAL protocol (Version V1.0, dated April 8, 2022).

## Data Availability

The datasets used and analyzed during the current study are available from the corresponding author on reasonable request.

## Abbreviations

ABPM: Ambulatory blood pressure monitoring
ADR: Adverse drug reaction
AE: Adverse event
CEC: Clinical endpoint committee
DBP: Diastolic blood pressure
FAS: Full analysis set
HBPM: Home blood pressure measurement
IDMC: Independent data monitoring committee
ITT: Intention to treat
LOCF: Last observation carried forward
OBPM: Office blood pressure measurement
PPS: Per-protocol set
SAE: Serious adverse event
SAP: Statistical analysis plan
SBP: Systolic blood pressure
SD: Standard deviation
SS: Safety set
TTR: Time in target range
ULN: Upper limit of normal
